# Improved CO_2_/CH_4_ Separation in Carbon Molecular Sieve Membranes via Copolymerization of Long-Chain Flexible Structures

**DOI:** 10.3390/membranes15050128

**Published:** 2025-04-27

**Authors:** Yingxiu Wu, Haiyan Guo, Bingyu Zhao, Yuxiu Yu, Yaodong Liu, Shouchun Zhang

**Affiliations:** 1 Shanxi Key Laboratory of Carbon Materials, Institute of Coal Chemistry Chinese Academy of Sciences, 27 Taoyuan South Road, Taiyuan 030001, China; wuyingxiu22@mails.ucas.ac.cn (Y.W.); zby20210810@163.com (B.Z.); yuyuxiu@sxicc.ac.cn (Y.Y.); 2 Center of Materials Science and Optoelectronics Engineering, University of the Chinese Academy of Sciences, Beijing 100049, China; 3 Research Group of New Energy Materials and Devices, State Key Laboratory of Coal and CBM Co-Mining, North University of China, Taiyuan 030051, China; sz202216017@st.nuc.edu.cn

**Keywords:** carbon molecular sieving membranes, gas separation, polyimide, PDMS, flexible structures

## Abstract

Carbon molecular sieve (CMS) membranes demonstrate considerable advantages and significant potential in the separation of CO_2_ and CH_4_. Nevertheless, current research predominantly emphasizes the enhancement of CMS membranes through the incorporation of rigid structures and chain spatial stacking. The role of flexible structures in this context remains inadequately understood. To address this gap, we introduced long-chain polydimethylsiloxane (PDMS) and copolymerized it to synthesize polyimide that combines rigid and flexible frameworks. This approach enabled us to investigate the impact of flexible structures on the structure and properties of carbon membranes by varying the PDMS content. The findings indicated that flexible PDMS significantly influenced the thermal decomposition behavior of polyimide and facilitated in situ silicon doping within the carbon membranes, thereby modifying the pore characteristics of the carbon film. Specifically, with a 10% addition of PDMS, the CO_2_ permeability of the CMS membrane reached 9556 Barrer, representing an enhancement of 103.9% and surpassing the 2019 upper bound for CO_2_/CH_4_ separation. Furthermore, the effect of pyrolysis temperature was also examined. Ultimately, this study offers a novel perspective on regulating the structural and performance characteristics of carbon membranes through the integration of long-chain flexible structures.

## 1. Introduction

Natural gas, recognized as a clean and efficient energy source, occupies a significant position within the global energy landscape. In recent years, the global consumption of natural gas has exhibited a pattern of fluctuating growth. Notably, during the first half of 2024, global natural gas demand experienced a substantial increase of approximately 3% compared to the previous year [1]. However, the presence of carbon dioxide (CO_2_) in natural gas can diminish its fuel calorific value and may lead to corrosion in transportation pipelines [2]. Consequently, the effective separation of CO_2_ and methane (CH_4_) in the fields of natural gas decarbonization and biogas purification is crucial for obtaining high-purity clean energy [3,4].

Membrane separation technology has gained considerable attention in gas separation due to its advantages, including low energy consumption, straightforward operation, and environmental friendliness [5,6,7]. To further enhance separation efficiency and reduce energy usage, the development of novel high-performance membrane materials is essential. Carbon molecular sieve (CMS) membranes, characterized by their rigid dual-channel structure, exhibit remarkable permeability or selectivity, which presents significant application potential [8,9,10]. Research indicates that the structure and performance of carbon molecular sieve membranes are heavily influenced by the precursor materials utilized [11,12,13,14,15]. Among these, polyimide has emerged as the predominant precursor for CMS membranes, particularly those polymerized from 4,4′-(Hexafluoroisopropyl)dibenzenedicarboxylic anhydride (6FDA)-based aldehydes. The 6FDA unit induces a helical conformation in polyimide chains, resulting in an increased free volume that contributes to the high permeability of the resultant CMS membranes [16]. Furthermore, rigid amine monomers such as (3,5-Diaminobenzoic acid) DABA and (Dimethylacetamide) DAM can also enhance permeability by increasing inter-chain spacing or forming cross-linked structures [17,18,19].

Nevertheless, studies have demonstrated that in addition to the spatial stacking of precursor chains, the thermal decomposition behavior of these chains significantly affects the pore structure of the carbon membranes [20,21]. For instance, the decomposition of 6F groups and methyl groups facilitates the formation of micropores or ultra-micropores within the carbon membranes [22]. Thus, introducing thermally less stable aliphatic hydrocarbon flexible chains into the precursor could potentially optimize the structure through spatial and thermal decomposition effects. Our previous research has indeed substantiated this premise. We found that incorporating 6FDA as the aldehyde monomer, along with copolymerization of rigid carboxylic groups and flexible siloxane segments, enabled a simultaneous enhancement of permeability and selectivity in CMS membranes [23]. Additionally, the spatial distribution of the copolymerized segments can be manipulated to fine-tune the structure and performance of the CMS membranes. The significant performance improvements have guided us to focus on the effects of flexible segments. In prior studies, we utilized short flexible chains; however, early investigations have indicated that the length of the flexible chains may also influence the structure of the CMS membranes [24,25]. Unfortunately, corresponding research into this mechanism remains limited.

Therefore, in this study, we employed long-chain siloxane flexible segments and copolymerized them with DABA and 6FDA to obtain polyimide precursors that incorporate both rigid and flexible structures, aimed at fabricating carbon molecular sieve membranes. By adjusting the content of the flexible segments, we aim to modulate the structure and performance of the carbon molecular sieve membranes. The influence of the long-chain siloxane flexible segments on the pore structure of the carbon membranes and their CO_2_/CH_4_ separation performance will be examined using characterization techniques such as X-ray diffraction (XRD), Raman spectroscopy, and pore size distribution (PSD). Additionally, we will investigate structural modulation by varying the pyrolysis temperature. This paper aspires to provide new insights and supplementary data on the structural control of flexible chains, ultimately facilitating the development of high-performance carbon molecular sieve membranes.

## 2. Materials and Methods

### 2.1. Materials

The 4,4′-(Hexafluoroisopropyl)dibenzenedicarboxylic anhydride (6FDA, purity ≥ 99%), poly(dimethylsiloxane)-bis(3-aminopropyl) capped (PDMS molecular weight 1000), and N-methylpyrrolidinone (NMP, purity ≥ 99.9%) were purchased from Aladdin Biochemistry Technology Co., Ltd., Shanghai, China. Analytically pure reagents such as 3,5-diaminobenzoic acid (DABA), 3-methylpyridine, acetic anhydride, and methanol were purchased from Tianjin Komeo Chemical Reagent Co., Ltd., Tianjin, China.

### 2.2. Synthesis of Polysiloxane-Based Polyimides (PIS)

**Figure 1:**
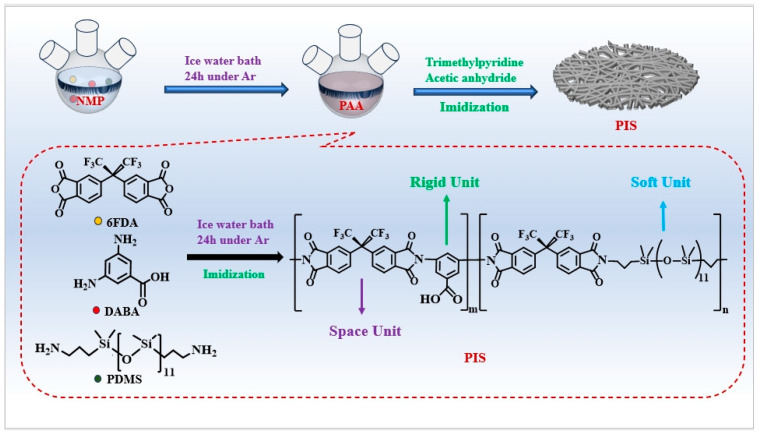
Scheme of synthesis for PISs.

In this experiment, a two-step method was employed for the synthesis of the PIS precursor [23]. As illustrated in Figure 1, the initial step involved the synthesis of polyamic acid. Prior to the addition of reagents, the three-necked flask was purged with argon in order to remove any residual air. Subsequently, the flask was cooled with an ice bath and 20 mL of NMP solvent was added to the 50 mL three-necked flask. The DABA:PDMS was then added to the NMP at mass ratios of 10:0, 9:1, 8:2, 7:3, and 6:4, respectively (The specific dosages are summarized in Table S1). Once the DABA and PDMS had been fully dissolved, the 6FDA powder was added to the solution in four discrete batches, with an interval of 10 min between each addition. Following a 20 min period during which the ice bath was in place, the reaction was continued at room temperature for a further 24 h, with the objective of obtaining a transparent polyamidoacid solution. In the second step, the above-synthesized polyamide acid solution was subjected to chemical dehydration to form polyimide via the chemical imide method. Specifically, 3-methylpyridine (0.97 mL) and acetic anhydride (9.45 mL) were added to the above solution in a sequential manner, resulting in the successful synthesis of PIS through imidization with stirring at room temperature for 24 h. Secondly, the phase conversion of the PIS solution was accomplished in a methanol solvent, and the resulting products were washed three times with methanol and then vacuum-filtered. The products were subsequently dried under vacuum at 120 °C for 24 h. The aforementioned synthesized products were designated PIS-0, PIS-1, PIS-2, PIS-3, and PIS-4, respectively, according to the content of PDMS.

### 2.3. Preparation of CMS Membranes

The preparation of the precursor membrane is as follows: Firstly, 0.1 g of PIS powder was dissolved in NMP solution to form a solution with a solid content of 10 wt%. This solution was then stirred at 60 °C for 12 h. Once complete dissolution had occurred, the solution was poured onto a clean glass plate in order to form a membrane by flow extension. The film was then subjected to a 24 h drying process at 60 °C in an oven. This was followed by a 12 h immersion in methanol with the objective of further removing any residual NMP from the film. Subsequently, the PIS precursor film was produced through vacuum drying at 120 °C for 24 h.

The preparation of carbon molecular sieve membranes is a multi-step process. PIS precursor membranes were placed in a tube furnace filled with argon gas for pyrolysis. The heating procedure of the tube furnace was as follows: from room temperature to 300 °C with a heating rate of 12 °C/min, from 300 °C to 370 °C with a rate of 5 °C/min, and then at a constant temperature of 60 min. Thereafter, the temperature was increased to 535 °C/585 °C/685 °C with a rate of 3 °C/min. The temperature was increased at a rate of 25 °C/min, reaching 535 °C/585 °C/685 °C. Thereafter, the temperature was increased at a rate of 0.25 °C/min to reach the final temperature of 550 °C/600 °C/700 °C. The temperature was maintained at this level for a period of 2 h. The temperature was increased to 685 °C at a rate of 0.25 °C/min, from 535 °C/585 °C/685 °C, and maintained at this level for 2 h [14]. The resulting carbon film was designated C-PIS.

### 2.4. Permeation Measurement

The permeability test of the membranes was conducted using the variable pressure constant volume method [26]. The downstream chamber volume of the test apparatus was 38.6 mL, the temperature of all the gases was 35 °C, and the feed pressure was 55 psi. The test gases were performed in the following order: CH_4_, H_2_, and CO_2_ and the membrane samples were degassed under vacuum for a minimum of six hours. All the membranes were tested on at least three samples. The formula for calculating the permeability of a single gas is shown in (1).

(1) $$P_{i}=\frac{273.15}{76}\frac{V}{AT}\frac{L}{∆p_{i}}(\frac{dp}{dt})$$

where *P* is gas permeability (Barrer, 1 Barrer = 1 × 10^−10^ cm^3^ (STP) cm cm^−2^ s^−1^ cmHg^−1^). The *V* is the downstream volume, the *T* is the test temperature and the Δ*p_i_* is the pressure difference. The *A* is the test area of the CMS membranes and *L* is the thickness of the membrane. The (*dp*/*dt*) is the slope of the pressure and time (s).

The ideal selectivity of the gas pair is determined by means of Equation (2).

(2) $$\alpha_{i/j}=\frac{P_{i}}{P_{j}}$$

where the *α* is the ideal selectivity of two gas molecules, and *P_i_* and *P_j_* represent the permeability of *i* gas and *j* gas, respectively.

### 2.5. Characterizations

The molecular structure and functional group information of PIS were obtained by 400 MHz narrow-cavity liquid nuclear magnetic resonance spectroscopy (NMR, AVANCE III, Bruker, Karlsruhe, Germany) and vacuum-type infrared emission spectrometry (ATR-FTIR, BrukerVERTEX 80v, Bruker, Karlsruhe, Germany), and field-emission scanning electron microscopy (SEM, JSM-7900F, Jeol, Tokyo, Japan) was used to observe the cross-section morphology and structure of PIS precursor membrane and carbon molecular sieve membrane cross-sectional morphology and structure; X-ray diffraction (XRD, Bruker D8 ADVANCE, Bruker, Karlsruhe, Germany) was performed using Ni-filtered CuKα radiation (λ = 0.154 nm) at the rate of 0.1◦/min to scan and characterize the samples; X-ray photoelectron spectroscopy (XPS, Kratos, Manchester, UK) was used to analyze the chemical state of the surface of the CMS membranes, which yielded C1s, Si2p peaks; thermogravimetric analysis (TGA, Q50, TA, Delaware, United States of America was used to study the thermal stability of the materials, and decomposition temperatures were characterized; a confocal Raman imaging system (Raman, WITec alpha300 RAS, WITec GmbH, Ulm, Germany) was used to characterize the degree of graphitization and defects of CMS membranes; and isothermal adsorption curves and pore size distributions of CMS membranes were obtained from CO_2_ isothermal adsorption tests using a specific surface area and pore size distribution meter (BET, V-Sorb Model 4800T, Ultrametrics, Beijing, China) at 273.15 K.

## 3. Results

### 3.1. Characterization of PISs

**Figure 2:**
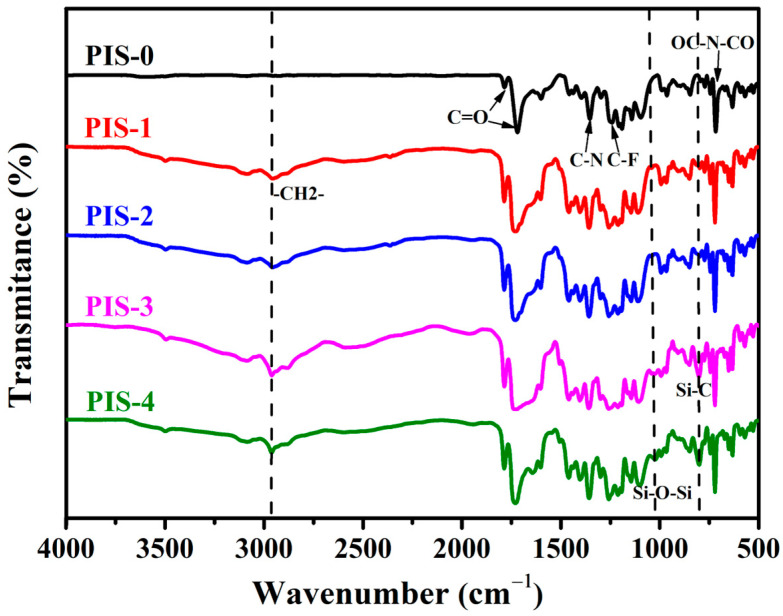
ATR-FTIR spectrum of PIS membranes.

The ATR-FTIR spectrum of PISs is presented in Figure 2. Prominent absorption peaks at 1784 cm⁻^1^ and 1716 cm⁻^1^ correspond to the asymmetric stretching vibrations of the C-O bond in the carboxyl functional group of DABA and the C-O bond in the imine carbonyl group, respectively. Additionally, the peaks at 1353 cm⁻^1^ and 721 cm⁻^1^ are attributed to the C-N and OC-N-CO vibrations, respectively, confirming the successful cyclization of polyamic acid to form an imine structure. The peaks observed at 1250 cm⁻^1^, 1203 cm⁻^1^, and 1150 cm⁻^1^ originate from the C-F bond within the 6FDA segment [27]. Moreover, the peak at 799 cm⁻^1^ is associated with the Si-C bond, with its intensity correlating positively with the increasing content of polysiloxane [28]. Notably, in comparison to the PIS-0 sample, the polyimide copolymerized with siloxane exhibited additional absorption peaks at 2960 cm⁻^1^ and 1038 cm⁻^1^; these are attributed to the asymmetric stretching of the R-CH_2_ group and the Si-O-Si bond, respectively. The intensities of these peaks were observed to increase with higher polysiloxane content. Collectively, the infrared spectra substantiate the successful copolymerization of polyimide with siloxane.

**Figure 3:**
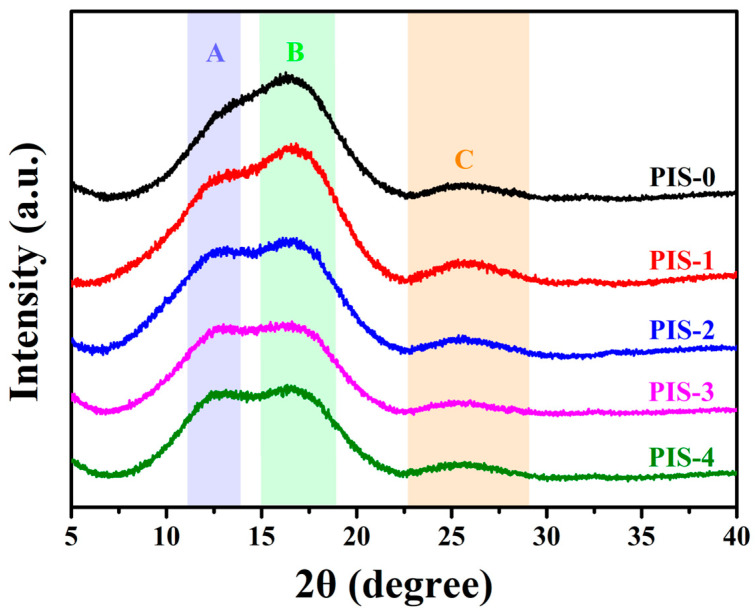
XRD patterns of PIS membranes. Peak A and Peak B are related to the chain stacking structure of the precursor membrane, Peak C is associated with the π-π stacking of the imide ring.

After preparing each polymer into a membrane, the cross-sectional SEM and elemental mapping images show that the Si element is uniformly distributed inside the membrane (Figure S1). When the PDMS content increases, a small amount of Si aggregation phase can be observed. The formation of the Si aggregation phase may be related to the interactions and intermolecular forces between PDMS segments. The interactions between PDMS segments are relatively weak, possibly due to the high bond energy of the silicon-oxygen bond (Si-O), which allows PDMS segments to have greater freedom in space. This weak interaction may lead to PDMS segments being more prone to approaching and aggregating together at high concentrations. Further investigation is needed into the effect of introducing flexible chains on the stacking of polyimide chains, as illustrated in Figure 3. The XRD spectra displayed complete amorphousness characterized by three broad peaks. Peaks A and B were identified at 12.5° and 16.5°, respectively, which are associated with the chain stacking of the precursor membranes. Peak C, occurring at 25°, is linked to the π-π stacking of the imide ring, as documented in the literature [29]. The progressive increase in the content of polysiloxane PDMS within the polymer resulted in a shift in Peak B to lower angles, indicating that the incorporation of flexible polysiloxane chains into the polyimide backbone enhanced the spatial freedom of the molecular chains, effectively modifying the stacking structure of the polyimide segments.

**Figure 4:**
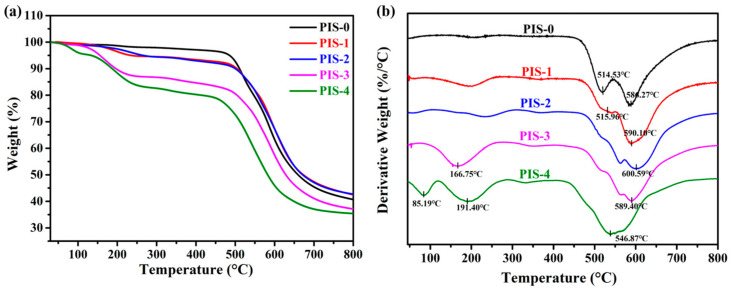
(**a**) TGA curves of PISs; (**b**) derivative weight loss curves.

The TGA results and derivative weight loss curves of the PIS polymer, as illustrated in Figure 4, reveal the thermal degradation characteristics of the materials. The TGA curve of the pure PI membrane demonstrates a consistent weight profile up to 450 °C, followed by a rapid decline occurring above 460 °C. The maximum weight loss temperatures are at 514 °C and 586 °C. The first maximum weight loss is primarily attributed to the decomposition of functional groups such as C-F and C=O, while the second maximum weight loss is mainly due to the cleavage of the polymer backbone [11]. Subsequently, the rate of weight loss diminishes at 700 °C, plateauing at 800 °C, with a total polymer weight loss of 60.95% recorded at 860 °C. Incorporating polysiloxane PDMS induced initial weight loss at approximately 200 °C, likely associated with the evaporation of residual solvents within the polymer matrix. From 200 °C onward, both PIS-1 and PIS-2 exhibited similar thermal degradation patterns, with no significant weight loss observed between 200 °C and 450 °C. Upon observing Figure 4b, it is found that as the PDMS content increases to 10%, 20%, and 30%, the first maximum weight loss temperature shifts to the right. This may be due to the oxidation of PDMS at high temperatures. However, when excessive PDMS is introduced, the second maximum weight loss temperature of the polymer (around 590 °C) begins to shift to the left. The main reason is that the excess PDMS may lead to phase separation. When the PDMS content is too high (40%), it may form independent PDMS phases within the PI matrix instead of being uniformly dispersed. Phase separation can result in the structural inhomogeneity of the material and disrupt the continuity of the internal structure. These defects can weaken the stability of the polyimide backbone, leading to a decrease in its decomposition temperature. The onset of thermal degradation for these samples occurred around 500 °C, with total weight losses of 59.32% and 58.60% for PIS-1 and PIS-2, respectively, at 860 °C. In contrast, PIS-3 and PIS-4 displayed three distinct phases of weight loss between 200 °C and 800 °C, with their decomposition temperatures being lower than those of PIS-1 and PIS-2. The final total weight losses for PIS-3 and PIS-4 were recorded at 64.11% and 66.87%, respectively. Therefore, when the content of PDMS is greater than or equal to 30%, the thermal stability of polyimide is significantly reduced. This phenomenon suggests that excessive PDMS may disrupt the aromatic char structure of PI, forming a loose silicon–oxygen–carbon network, thereby reducing high-temperature stability, and the thermal stability of polyimide is significantly reduced due to the violent decomposition of flexible chains.

### 3.2. Characterization of CMS Membranes

**Figure 5:**
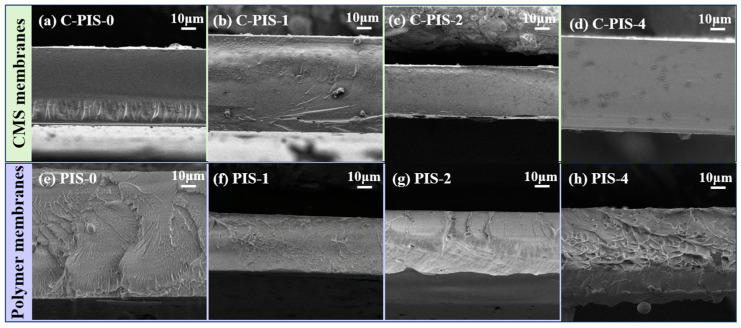
(**a**–**d**) Cross-sectional SEM images of CMS membranes; (**e**–**h**) cross-sectional SEM images of polymeric membranes.

Upon carbonization at 550 °C, the scanning electron microscopy (SEM) analysis of the membranes’ cross-section revealed that the carbon molecular sieve (CMS) membranes demonstrated a denser and more uniform morphology compared to the polymer membranes (Figure 5). Notably, the cross-section of the CMS membranes exhibited increased roughness with the incorporation of PDMS relative to C-PIS-0. Particularly at elevated concentrations, the aggregation of PDMS led to the formation of dark-colored regions within the cross-section of C-PIS-4, suggesting the retention of silicon from PDMS within the carbon membranes post-carbonization [30]. Additionally, the Si2p fine spectrum confirmed the presence of silicon in C-PIS-1, 2, 3, and 4, indicating that silicon is integrated into the carbon membranes in the form of Si-C and Si-O bonds (Figure S2).

**Figure 6:**
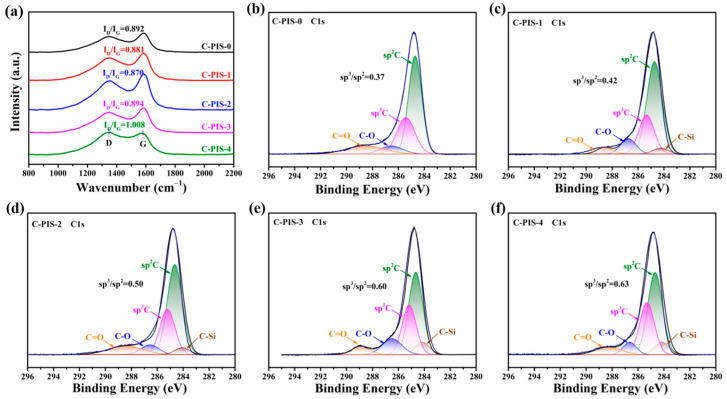
The characterization of carbon structure for CMS membranes carbonized at 550 °C. (**a**) Raman spectra and (**b**–**f**) C1s spectra of C-PIS.

Raman spectroscopy was utilized to investigate the effects of varying PDMS concentrations on the structural properties of the CMS membranes, as shown in Figure 6a. The CMS membranes presented distinct broad peaks at approximately 1350 cm⁻^1^ (D peak) and 1580 cm⁻^1^ (G peak), respectively [31]. The D peak corresponds to the A_1g_ symmetric telescopic vibrational mode, indicating an increased quantity of sp^3^-hybridized carbon atoms in the carbonized membranes. This increment reduces the electron cloud density within the graphite-like network, thereby weakening the π-π interactions between the graphite layers, resulting in an expanded interlayer distance and the formation of additional micropores. Conversely, the G peak, associated with the in-plane stretching vibration of sp^2^-hybridized carbon, suggests the presence of a more ordered graphitic carbon structure composed of a greater amount of microcrystalline graphite [32]. The detection of both peaks confirms that the synthesized CMS membranes possess a graphite-like structure, characterized by a coexistence of disordered amorphous graphite and ordered microcrystalline graphite. The peak height ratio of D to G (I_D_/I_G_) serves as a metric for assessing the defect density within the carbon molecular sieve membranes. The computed I_D_/I_G_ values for C-PIS-0, C-PIS-1, C-PIS-2, C-PIS-3, and C-PIS-4 were 0.892, 0.881, 0.870, 0.894, and 1.008, respectively. The nearly constant I_D_/I_G_ ratio suggests that PDMS exerts a minimal influence on the graphitic structure of the carbon film. The XRD analysis reveals that all the carbon films exhibit an amorphous structure (Figure S3). This finding indicates that the internal architecture of the carbon membrane significantly deviates from the ordered arrangement characteristic of graphite. Koros et al. reported that following the carbonization of polyimide at 550 °C, the microstructure of the carbon membrane comprises disordered carbon chains, along with a limited number of carbon sheet structures [25]. Consequently, the structural evaluation based solely on the D and G peaks is insufficient to accurately characterize the prevalent amorphous carbon chains or carbon flakes.

To further elucidate the influence of PDMS on the carbon structure of the films, the C1s fine spectrum was analyzed (Figure 6b–f). The results confirm the presence of sp^2^ carbon, sp^3^ carbon, C-O, and C=O functional groups in all the membrane structures. Notably, with the incorporation of PDMS, an additional C-Si component is observed. Furthermore, the introduction of PDMS impacts the ratios of sp^2^ to sp^3^ carbon; as the concentration of PDMS increases, the proportion of sp^3^ carbon relative to sp^2^ carbon also rises. This trend indicates an enhancement in defect-laden carbon structures, which may significantly influence the gas separation performance of the carbon membranes.

**Figure 7:**
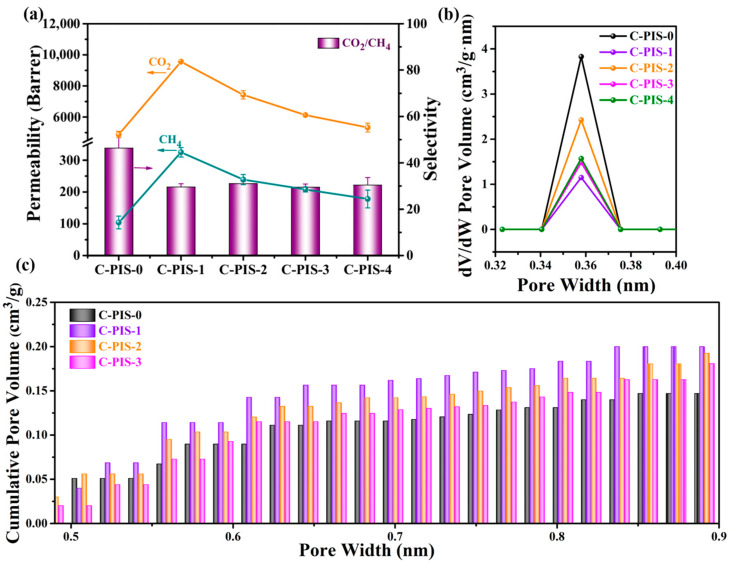
(**a**) Single gas separation performances of CMS membranes carbonized at 550 °C (C-PIS-0, 1, 2, 3, and 4); (**b**) the PSD change in ultra-micropores for C-PIS-0, 1, 2, 3, and 4 from CO_2_ sorption at 273 K; (**c**) cumulative pore volume for C-PIS-0, 1, 2, and 3.

Figure 7a presents a comprehensive summary of the pure gas separation performance of polyimide membranes following carbonization at 550 °C. Detailed values are listed in Table 1, revealing the permeability ranking for various gases as follows: P(H_2_) > P(CO_2_) > P(CH_4_), which corresponds inversely to their kinetic diameters. The permeability of carbon dioxide and methane for C-PIS-0 are recorded at 4882 Barrer and 104 Barrer, respectively. The CO_2_/CH_4_ selectivity for C-PIS-0 is determined to be 46.94. The incorporation of PDMS at a concentration of 10% significantly enhanced the permeability of the resulting carbon membrane, designated as C-PIS-1. In this context, the observed fluxes are 11,200 Barrer for H_2_ and 9556 Barrer for CO_2_, indicating enhancements of 69.7% and 103.9% in permeability for H_2_ and CO_2_, respectively, compared to C-PIS-0. The reason for this lies in the pyrolysis process, where the aromatic imine structural domains in PIS are converted into a carbon-rich phase, functioning as a selective phase for gas molecules. Meanwhile, the PDMS domains within the PIS matrix transform into a silicon-rich phase containing small carbon clusters, thereby enhancing gas permeability. The presence of longer PDMS chains leads to the formation of PDMS structural domains, which exhibit a microphase-separated structure composed of imine and siloxane blocks. As a result, variations in the composition of these two phases contribute to distinct gas separation performances. However, a notable limitation of the study is that C-PIS-1 demonstrates a trade-off effect between permeability and selectivity, resulting in a CO_2_/CH_4_ selectivity of 29.4. Further increases in PDMS concentration led to a substantial reduction in gas permeability of the carbon membrane substructures without a corresponding improvement in CO_2_/CH_4_ selectivity. This could be because when the PDMS content is too high, the interactions between the chain segments are enhanced, leading to a decrease in the mobility of the chain segments. At the same time, the reduction in free volume also narrows the diffusion channels for gas molecules within the PDMS, increasing the diffusion resistance. These factors collectively contribute to a decrease in gas permeability.

The pore structure of CMS membranes elucidates the effects of varying PDMS concentrations on the gas separation performance of polyimide. As depicted in Figure 7b, pore size distributions (PSDs) of the CMS membranes are presented. We utilized the Non-Local Density Functional Theory (NLDFT) to fit the measured CO_2_ adsorption–desorption data, and the distribution of ultra-micropores with diameters less than 0.4 nm is shown in Figure 7b. These ultra-micropores are primarily concentrated at 0.358 nm, a size that is intermediate between the kinetic diameters of CO_2_ (0.33 nm) and CH_4_ (0.38 nm), thus enabling direct molecular sieving effects. Smaller CO_2_ molecules can pass through more rapidly, while larger CH_4_ molecules experience stronger retention and are hindered from passing through the carbon molecular sieve membrane. The study revealed that the variation in the volume of the smallest micropores among the five PIS-based carbon molecular sieve membranes follows the order of C-PIS-0 > C-PIS-2 > C-PIS-4 > C-PIS-3 > C-PIS-1, which is consistent with the gas selectivity for CO_2_/CH_4_. The cumulative pore volume of the CMS membrane is shown in Figure 7c. The PI-based CMS membrane without PDMS exhibits the smallest cumulative pore volume. With the introduction of PDMS into the polyimide precursor, the pore volume of the CMS membrane shows a significant increasing trend, providing more diffusion channels for gas molecules. Among them, the C-PIS-1 sample with a PDMS content of 10% exhibits the largest pore volume characteristics, and its excellent pore structure directly leads to the best gas permeability performance. However, it is worth noting that as the PDMS content continues to increase, the pore volume of the carbon membrane instead shows a decreasing trend. This phenomenon can be attributed to the excessive decomposition of flexible chains during the pyrolysis process caused by the high PDMS content, and the decomposition products cause a certain degree of damage to the pore structure, leading to pore collapse. Therefore, compared to the C-PIS-1 and C-PIS-2 samples with lower PDMS contents, the C-PIS-3 and C-PIS-4 samples with higher PDMS contents exhibit relatively poorer gas permeability performance.

Polyimide-based precursors are polymer materials with excellent thermal stability, and their pyrolysis process is a complex and orderly chemical reaction. During this process, the molecular structure of polyimide undergoes significant changes, ultimately forming a carbon molecular sieve membrane with a microporous structure. The pyrolysis temperature has a significant impact on the structure of the carbon membrane and its gas separation performance. The medium-temperature range (550 °C to 700 °C) is a relatively ideal temperature range for the pyrolysis of polyimide-based precursors to form carbon molecular sieve membranes. Within this temperature range, the precursors can undergo sufficient pyrolysis reactions to form carbon membranes with abundant microporous structures. At the same time, this temperature range also avoids the damage or sintering of the carbon membrane structure caused by excessively high temperatures. Consequently, PIS-1 was selected as the precursor membrane. CMS membranes were prepared at the pyrolysis temperatures of 550 °C, 600 °C, and 700 °C, respectively (denoted as C550-PIS-1, C600-PIS-1, and C700-PIS-1), to elucidate the impact of carbonization temperature on the gas separation performance of CMS membranes. Figure S4 illustrates the cross-sectional SEM images of C550-PIS-1, C600-PIS-1, and C700-PIS-1, respectively. The images demonstrate that these carbon membranes are characterized by a smooth and dense structure, devoid of any discernible split-phase pore structure.

**Figure 8:**
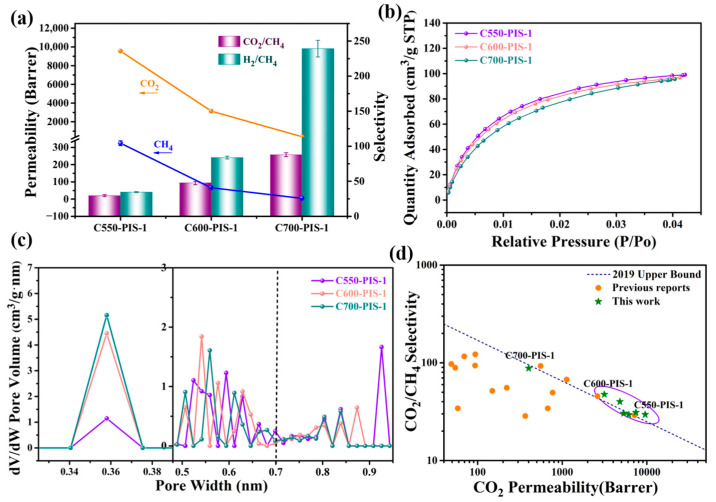
(**a**) Single gas separation performances of PIS-1 carbonized at different temperatures; (**b**) CO_2_ sorption isotherms measured at 273 K; (**c**) the PSD change in ultra-micropores and micropores; (**d**) a comparison of the CO_2_/CH_4_ separation of the current work with previous reports. The latest Robeson upper-bound lines are plotted for comparison.

The separation performance of individual gases is presented in Figure 8a. Detailed values are listed in Table 2, where the experimental observations indicate that an increase in pyrolysis temperature leads to reduced permeability but enhanced selectivity. When the pyrolysis temperature was raised to 600 °C, the permeability of CO_2_ was measured at 3144 Barrer, concurrently with a significant increase in the CO_2_/CH_4_ selectivity to 47.6. Elevating the pyrolysis temperature further to 700 °C resulted in CO_2_ and H_2_ permeabilities of 402 Barrer and 1096 Barrer, respectively. Simultaneously, the permeability of CH_4_ dramatically decreased from 325 Barrer at 550 °C to 4.58 Barrer. The selectivities for CO_2_/CH_4_ and H_2_/CH_4_ achieved remarkable values of 87.8 and 239.3, corresponding to increases of 198.5% and 594.2%, respectively, compared to those of C550-PIS-1. These substantial changes in gas separation performance are attributed to the influence of pyrolysis temperature on the ultra-microporous distribution.

In this study, the CO_2_ adsorption tests were conducted on PIS-1 at 273.15 K across various temperatures, with the resulting CO_2_ adsorption isotherms illustrated in Figure 8b. The adsorption capacities for the samples ranked as follows: C550-PIS-1, C600-PIS-1, and C700-PIS-1, corroborating the observed variations in CO_2_ permeability among these samples. The pore size distribution, analyzed using the Density Functional Theory model, is depicted in Figure 6c. When the pore size was less than 0.38 nm, the pore volume change was the largest for C700-PIS-1 and the smallest for C550-PIS-1, which directly accounted for the selectivity ranking of C700-PIS-1 > C600-PIS-1 > C550-PIS-1. When the pore size ranged from 0.5 nm to 0.9 nm, as the pyrolysis temperature increased, the distributions within both the microporous and ultra-microporous regions shifted towards smaller pore sizes. Specifically, as the temperature was raised from 550 °C to 700 °C, the PIS-1-based CMS membranes exhibited a notable shift in its ultra-microporous distribution from 0.55 nm to 0.5 nm and in the microporous region from 0.925 nm to 0.85 nm. This alteration in pore distribution indicates a decline in permeability accompanied by an increase in selectivity for all gases and gas pairs, consistent with the previously discussed gas permeability and selectivity data. These outcomes suggest that higher pyrolysis temperatures foster a more compact arrangement of carbon chains within the microporous walls.

Figure 8d presents a comparison of the CO_2_/CH_4_ separation performance from this study against the 2019 upper limit of CO_2_/CH_4_ and other CMS membranes. Detailed data can be found in Table 3. Notably, C550-PIS-1, C600-PIS-1, and C550-PIS-2 all surpassed the 2019 upper limit threshold. Compared to the previously reported results, C550-PIS-1 exhibited outstanding permeability, while C600-PIS-1 demonstrated satisfactory permeability coupled with enhanced selectivity. The remaining samples, C700-PIS-1, C550-PIS-3, and C550-PIS-4, are in close proximity to the 2019 upper limit.

## 4. Conclusions

In this study, 6FDA, DABA, and PDMS were copolymerized to synthesize a polyimide that incorporates both rigid and flexible structures. The structural properties and characteristics of the carbon film were modulated by varying the concentration of the flexible chains. The findings reveal that with a PDMS content of 10%, the degradation of the flexible chains resulted in an increased pore size of the carbon membrane, leading to a CO_2_ permeability of 9556 Barrer. Concurrently, this modification caused a reduction in CO_2_/CH_4_ selectivity, which surpasses the upper bound of 2019. However, upon further increasing the PDMS content, the continued degradation of the flexible chains induced the structural collapse of the carbon film. This collapse resulted in a reduction in pore density, with minimal changes to pore size, ultimately diminishing CO_2_ permeability while preserving CO_2_/CH_4_ selectivity. These observations underscore the significant influence of flexible chain thermal decomposition on the pore architecture of carbon films. Additionally, the impact of carbonization temperature on the properties of the carbon membranes was investigated. The results indicate that as the carbonization temperature escalates, both pore density and size decrease, leading to reduced CO_2_ permeability. Conversely, CO_2_/CH_4_ selectivity is markedly enhanced at higher temperatures. Therefore, this study elucidates how the thermal decomposition of flexible chains can optimize the separation performance of carbon membranes, offering valuable insights into the structural regulation of these materials.

## Figures and Tables

**Table 1:** Gas separation properties of PIS carbonized at 550 °C. Membrane thicknesses are about 50 μm.

Sample	Permeability (Barrer)			Ideal Selectivity	
	CH_4_	CO_2_	H_2_	CO_2_/CH_4_	H_2_/CH_4_
C-PIS-0	104 ± 20	4880 ± 200	6600 ± 200	46.9 ± 9.2	64 ± 12
C-PIS-1	325 ± 15	9556 ± 10	11,200 ± 330	29.4 ± 1.4	34.5 ± 1.9
C-PIS-2	239 ± 16	7420 ± 270	7300 ± 330	31.1 ± 2.4	30.6 ± 2.5
C-PIS-3	208 ± 8	6124 ± 66	7166 ± 59	29.4 ± 1.2	34.5 ± 1.4
C-PIS-4	178 ± 28	5320 ± 290	6900 ± 300	30.0 ± 5.0	38.8 ± 6.3

**Table 2:** Gas separation properties of PIS-1 carbonized at different temperatures.

Sample	Permeability (Barrer)			Ideal Selectivity	
	CH_4_	CO_2_	H_2_	CO_2_/CH_4_	H_2_/CH_4_
C550-PIS-1	325 ± 15	9556 ± 10	11,200 ± 330	29.4 ± 1.4	34.5 ± 1.9
C600-PIS-1	66 ± 2	3144 ± 47	5560 ± 320	47.6 ± 1.6	84.3 ± 5.5
C700-PIS-1	4.58 ± 0.07	402 ± 6	1096 ± 28	87.8 ± 1.9	239.3 ± 7.1

**Table 3:** Comparison of gas separation performances of CMSMs from this work and the literature.

Precursors	Carbonization Temperature (°C)	CO_2_ Permeability (Barrer)	CH_4_ Permeability (Barrer)	Selectivity of CO_2_/CH_4_	Ref.
6FDA-6FpDA/DABA	576	2610	57.3	45.55	[21]
6FDA-6FpDA/DABA	700	771	15.6	49.42	[21]
6FDA-6FpDA/DABA	800	221	3.99	55.39	[33]
PIM-6FDA-OH	600	5450	132	41.29	[33]
PIM-6FDA-OH	630	2870	58	49.48	[33]
PIM-6FDA-OH	800	556	6	92.67	[33]
PFA/CNT/AAO	600	69.57	/	116.23	[15]
LC-600-298K	600	572	16	35.75	[34]
PC-600-298K	600	49.54	1.45	34.17	[34]
TC-0-2	600	621	21.8	28.5	[35]
TC-1-2	600	253	4.9	51.6	[35]
TC-2-2	600	83.1	0.85	97.3	[35]
TC-3-2	600	92.5	1.04	88.9	[35]
6FDA/BPDA-DAM(1:1)	550	7170	247	29.03	[36]
6FDA/BPDA-DAM	550	1280	18.5	69.19	[37]
6FDA/BPDA-DAM	800	93.5	0.997	93.78	[37]
6FDA/BPDA-DAM	800	94	0.77	122.08	[37]
6FDA/BPDA-DAM(1:1)	675	1130	16.8	67.26	[38]
C-PIS-1	550	9556	325	29.4 ± 1.4	This work
C-PIS-1	600	3144	66	47.6 ± 1.6	This work
C-PIS-1	700	402	4.58	87.8 ± 1.9	This work
C-PIS-2	550	7421	239	31.1 ± 2.4	This work

## Data Availability

The data supporting the findings of this study are available within the article.

## Supplementary Materials

The following supporting information can be downloaded at https://www.mdpi.com/article/10.3390/membranes15050128/s1, detailed information of experimental sections (Supporting Information S1–S3), Table S1: The experimental information for the preparation of precursors; Figure S1: The cross-sectional SEM and EDS images of PIS; Figure S2: (a,b) Si2p spectra of C-PIS-1,2,3 and 4; Figure S3: XRD patterns of C-PIS; Figure S4: (a–c) Cross-sectional SEM images of CMS membranes carbonized at different temperatures.
