# Supplementary material for: Improved CO2/CH4 Separation in Carbon Molecular Sieve Membranes via Copolymerization of Long-Chain Flexible Structures
Source: Membranes (Basel). 2025 Apr 27;15(5):128. doi: 10.3390/membranes15050128 (PMC12113629; doi:10.3390/membranes15050128)
Supplement: Supplementary file 1 [file membranes-15-00128-s001.zip › membranes-3568350-supplementary.pdf]

Supporting Information

Improved CO_2/CH_4 Separation in Carbon Molecular Sieve Membranes via Copolymerization of Long-Chain Flexible Structures

Yingxiu Wu[1,3], Haiyan Guo[4], Bingyu Zhao[1,3], Yuxiu Yu[1], Yaodong Liu[1,2,*] and Shouchun Zhang[1,2,*]

[1] Shanxi Key Laboratory of Carbon Materials, Institute of Coal Chemistry Chinese Academy of Sciences, 27 Taoyuan South Road, Taiyuan 030001,China

[2] Center of Materials Science and Optoelectronics Engineering, University of the Chinese Academy of Sciences, Beijing 100049, China

[3] University of Chinese Academy of Sciences, 19 Yuquan Road, Beijing 100049,China

[4] Research Group of New Energy Materials and Devices, State Key Laboratory of Coal and CBM Co-Mining, North University of China, Taiyuan 030051, China

* **Corresponding author:** Yaodong Liu. (liuyd@sxicc.ac.cn) & Shouchun Zhang. (zschun@sxicc.ac.cn)

Table S1. The experimental information for the preparation of precursors.

Precursors	PDMS(μL)	DABA(g)	6FDA(g)	NMP(mL)
PIS-0	0	1.2172	3.5539	20
PIS-1	138	1.2172	3.6140	20
PIS-2	315	1.2172	3.6891	20
PIS-3	540	1.2172	3.7857	20
PIS-4	841	1.2172	3.9144	20

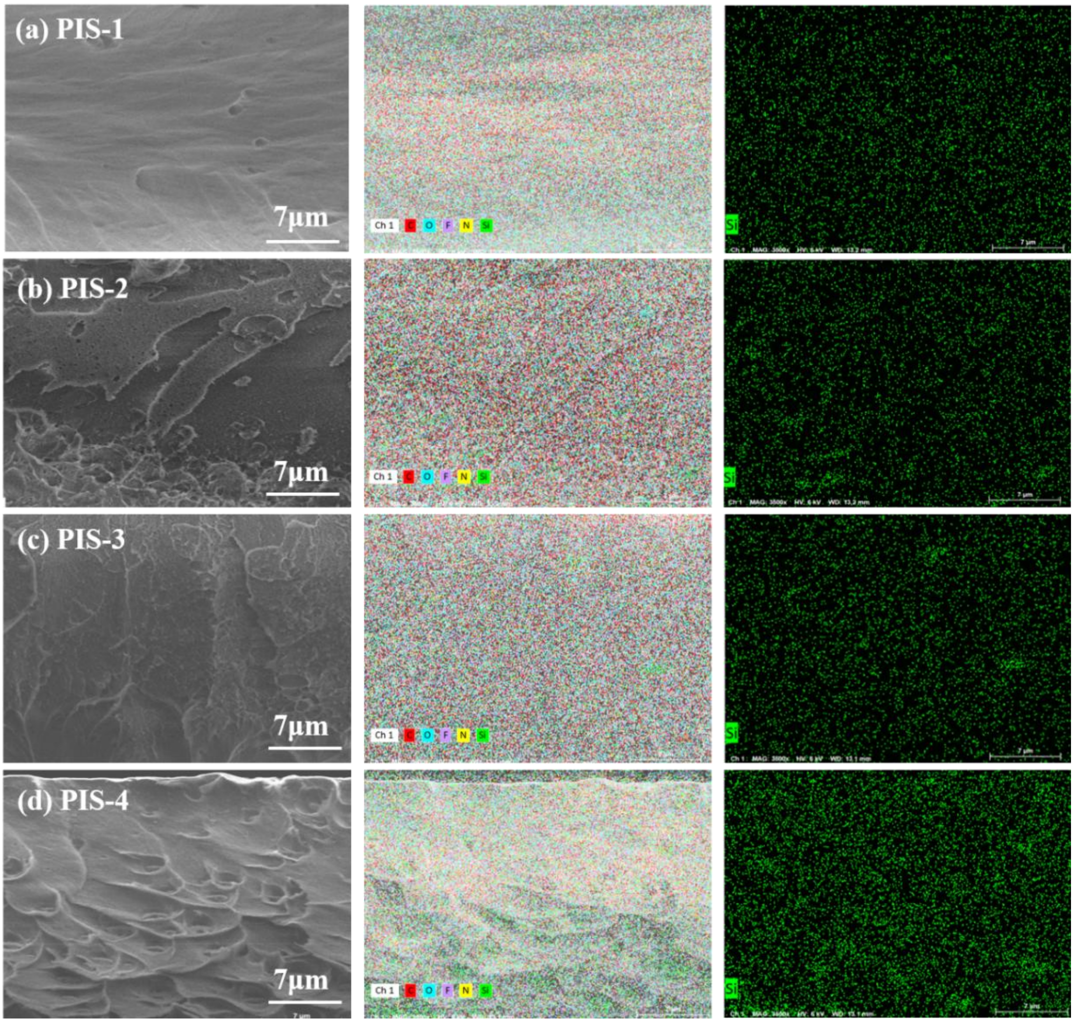

Figure S1. The cross-sectional SEM and EDS images of PIS.

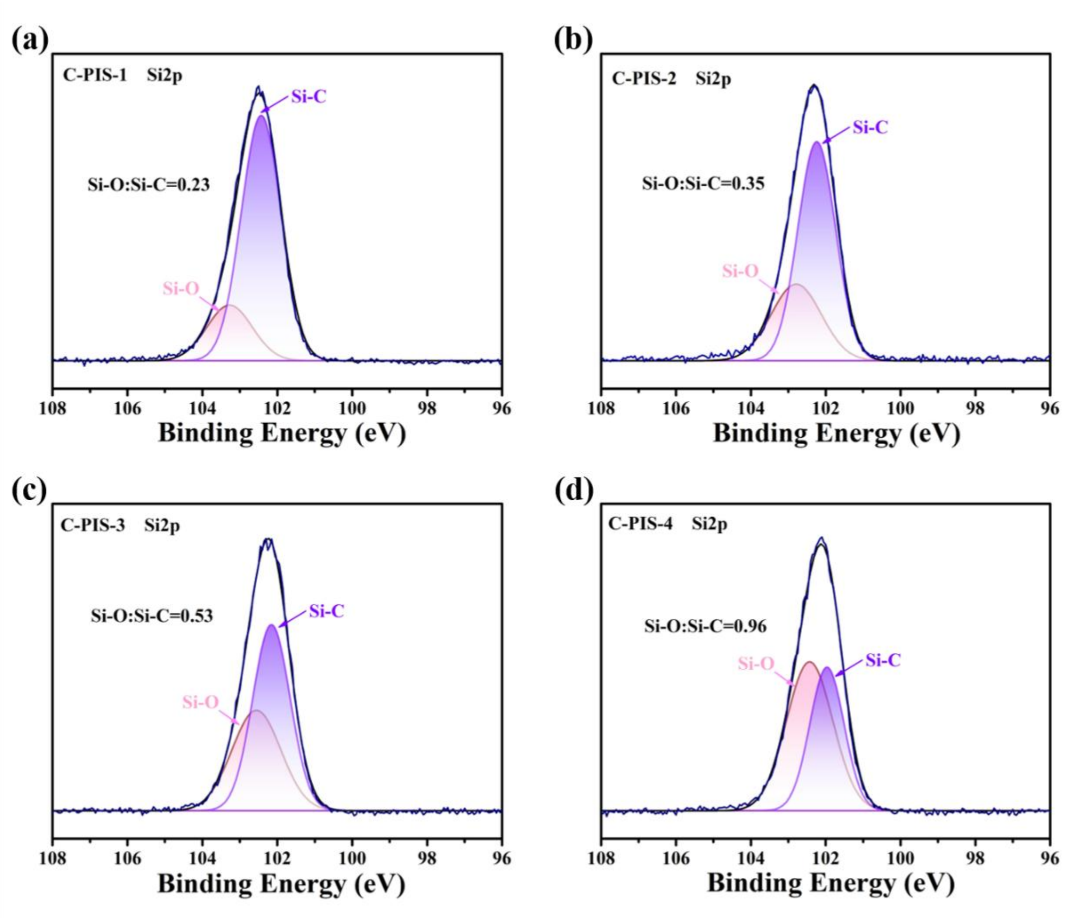

Figure S2. (a)-(b) Si2p spectra of C-PIS-1,2,3 and 4.

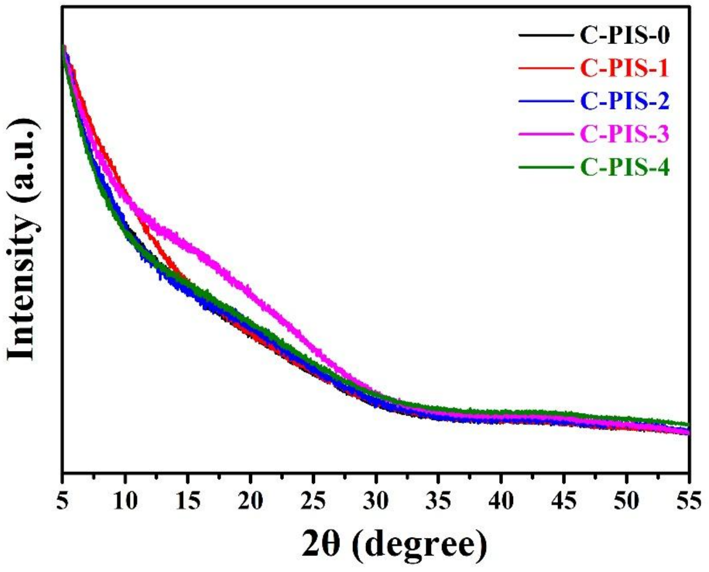

Figure S3. XRD patterns of C-PIS.

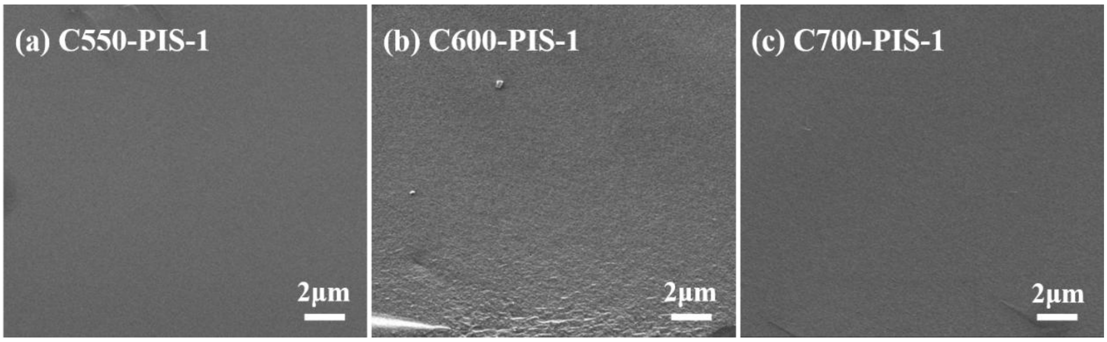

Figure S4. (a)-(c) Cross-sectional SEM images of CMS membranes carbonized at different temper-atures.
